# International Commission on Trichinellosis: Recommendations on pre-harvest control of *Trichinella* in food animals

**DOI:** 10.1016/j.fawpar.2019.e00039

**Published:** 2019-03-18

**Authors:** H. Ray Gamble, Lis Alban, Dolores Hill, Dave Pyburn, Brad Scandrett

**Affiliations:** a National Academy of Sciences, 500 Fifth Street NW, Washington, DC 20001, United States of America; b Danish Agriculture & Food Council, Axeltorv 3, DK-1609 Copenhagen, Denmark; c United States Department of Agriculture, Agricultural Research Service, Animal Parasitic Diseases Laboratory, Beltsville, MD 20705, United States of America; d National Pork Board, 1776 NW 114th St., Clive, IA 50325, United States of America; e Centre for Food-borne and Animal Parasitology, Canadian Food Inspection Agency, 116 Veterinary Road, Saskatoon, SK S7N 2R3, Canada

**Keywords:** *Trichinella*, Pork, Negligible risk, Controlled management

## Abstract

Transmission of *Trichinella* to domestic livestock, notably pigs, is limited to certain risk factors including feeding of raw meat-containing waste products or animal carcasses and exposure to infected rodents and wildlife. Prevention of infection in food animals is dependent on eliminating risk of exposure to these potential sources of *Trichinella.* By implementing conditions of controlled management, pig herds can be considered to pose a negligible risk for *Trichinella*, and groups of herds which follow these same conditions can be considered to be a compartment with negligible risk for *Trichinella*. Pork from pigs originating from a negligible risk herd or compartment would not require further testing or processing to protect consumers from this parasite. Verifying the status of pigs from a controlled management negligible risk herd or compartment can be accomplished by a program of regular audits or by implementing surveillance testing of a statistical sample of pigs from the herd or compartment.

## Highlights

• Exposure of pigs to *Trichinella* can be limited in a controlled management system.
• Controlled management prevents contact with wildlife, birds and rodents.
• Controlled management requires good manufacturing and storage practices for feed.
• Verification of controlled management can be achieved by auditing or surveillance.
• Pork considered negligible risk does not require testing or processing for *Trichinella*.

## 1 Introduction

*Trichinella* infection in domestic pigs is an extremely rare occurrence in modern pork production systems, as has been demonstrated in many countries by extensive testing of pig carcasses at slaughter. For that reason, it no longer makes sense to incur the expense of testing individual carcasses. In this recommendation, we describe and reference the conditions for pork production necessary to assure very low risk for exposure of pigs to *Trichinella*, and the processes of verification and documentation that can be referenced by veterinary and food safety authorities.

Infection with *Trichinella* spp. can only occur if an animal or human ingests muscle tissue containing infective larvae. Therefore, transmission of *Trichinella* to domestic livestock, notably pigs, is limited to certain well-documented risk factors including feeding of raw meat-containing waste products or animal carcasses and exposure to infected rodents and wildlife. Prevention of infection in food animals is dependent on eliminating risk of exposure to these potential sources of *Trichinella* (see Gamble et al., 2007 for a review)*.*

Preventing exposure to these sources of *Trichinella* infection requires that animals be raised in accordance with principles of controlled management, which includes controlled housing systems. When a production site reduces or eliminates risks of exposure to sources of *Trichinella* through controlled management, animals from that production site are considered to pose a negligible risk for public health purposes. Pigs reared in pork production systems which meet the conditions of controlled management, as described below, satisfy the criteria for classification as being of negligible risk and therefore individual slaughter inspection of animals raised under these conditions is not necessary for purposes of protecting public health and trade. When a group of pork production sites satisfy the criteria for controlled management, the animal population comprised thereof may be considered to represent a compartment of negligible risk.

The International Commission on Trichinellosis (ICT) considers the individual pork production site, or a group of pork production sites (a compartment), as the epidemiological unit in which is it possible to implement effective measures to prevent exposure of domestic pigs to *Trichinella*. Where controlled management is implemented to prevent exposure to *Trichinella*, as described here and in related documents (World Organization for Animal Health (2018a) and the Codex Alimentarius (2015), there is a negligible public health risk from consuming pork. When controlled management is implemented and routinely verified, there is sufficient scientific justification that individual testing of pig carcasses (at slaughter) for *Trichinella* infection should not be required.

## 2 Requirements for production of pigs with a negligible risk for *Trichinella*

The specific requirements of controlled management that need to be met for pigs to be considered to pose a negligible risk for *Trichinella* are as follows:

### 2.1 Controlled housing

Swine housing includes physical barriers which prevent swine from being exposed to wildlife (including birds) and carrion derived thereof and which greatly reduces swine from being exposed to rodents.

### 2.2 Feed and feed storage

Feed, and feed components, are stored in closed silos or containers, which do not attract or allow rodents or wildlife to enter. Feed which is not produced on-site is purchased from an approved facility, which conforms to good production practices (for example, see World Organization for Animal Health, 2018b). Waste food, if fed to pigs, must not contain meat products.

### 2.3 Rodent control

A documented rodent control program is maintained by a pest control provider or an employee(s) of the production facility and regular inspection for rodent activity is performed. When properly performed and documented, there should be no evidence of active rat infestations.

### 2.4 Farm hygiene

Dead animals are promptly removed from the pig housing areas and disposed of off-site or stored in animal-proof containers for removal from premises.

### 2.5 Introduction of new pigs

New animals, excluding piglets under five weeks of age, entering the production site must originate from farms that also apply controlled management conditions and have attained *Trichinella* negligible risk status.

### 2.6 Animal identification and traceability

Documentation exists so that movement of pigs and/or lots can be traced.

## 3 Negligible risk herds and compartments

Pigs from production systems that adhere to the controlled management requirements described here will have a negligible risk for exposure to *Trichinella*. Individual pork production sites meeting these requirements may be grouped together into a compartment, and all pigs from that compartment may be considered to have negligible risk of infection with *Trichinella*.

Verification and/or certification of the negligible risk status of a production site or compartment may be required for purposes such as food safety, marketing, or trade.

A general framework for assuring the integrity of a negligible risk production site or compartment should include a system for collecting and reviewing documentation demonstrating compliance with the requirements for controlled management. Documentation may include records kept during production (e.g., rodent control, animal movement records) and audits of the conditions of controlled management.

Regular review of documentation and auditing of production sites or compartments following the conditions of controlled management should facilitate compliance verification. Auditing and verification should include oversight of the process (i.e., a program of on-farm audits such as described by the World Organization for Animal Health (2018a) or oversight of the product (i.e., a program of surveillance testing demonstrating absence of infection at a level of risk that is acceptable for protection of public health; Codex, 2015). For the purposes of certifying negligible risk for a production site or compartment, auditing and surveillance may be used alternatively.

## 4 Verifying and certifying compliance with controlled management by auditing

Auditing is one method to assure that the conditions of controlled management are followed on production sites and in compartments establishing or maintaining a designation of having negligible risk for *Trichinella* infection. Audit design and audit frequency should be risk-based, taking into account historical information, pig management systems, slaughterhouse monitoring results, knowledge of established farm management practices and the presence of susceptible wildlife. Audits should address each element of controlled management as described in these recommendations and elsewhere.

If the purpose of auditing is to certify negligible risk, the auditing process should be overseen by the competent authority. Auditing should be performed by qualified and trained auditors, according to national or international standards. A regular schedule of audits of production sites/compartments participating in programs of controlled management should be implemented, including unannounced spot audits as appropriate.

## 5 Verifying and certifying compliance with controlled management by surveillance

Surveillance is another method for assuring that the product of controlled management meets established criteria for having negligible risk for *Trichinella* infection. The design prevalence for surveillance of a negligible risk production site/compartment should support a level of risk that is acceptable for protection of public health.

Surveillance programs for *Trichinella* infection should take into account the provisions of the World Organization for Animal Health (2018c), which states “surveillance is aimed at demonstrating the absence of disease or infection” and “surveillance is a tool … to provide data for use in risk assessment, for public health purposes, and to substantiate the rationale for sanitary measures.” In that regard surveillance for *Trichinella* in pigs within a defined epidemiological unit or population (e.g., compartment) can be used to determine public health risk.

### 5.1 Defining an acceptable level of risk for verifying controlled management

Guidelines from appropriate international authorities should be used to determine a level of risk to consumers that is acceptable to establish a program for surveillance of animals produced under controlled management conditions.

As an example, the Codex Alimentarius Committee on Food Hygiene (Codex, 2015) considers that “a slaughter surveillance programme [for *Trichinella*] incorporating current testing data demonstrating that prevalence of infection does not exceed 1 infected carcass per 1,000,000 pigs slaughtered with at least 95% confidence” will provide adequate assurance that pigs from a negligible risk compartment truly pose a negligible risk to consumers.

Consumer risk is dependent on a number of factors including prevalence of infection, test sensitivity of meat inspection, larval distribution in infected carcasses, culinary habits and per capita consumption of pork. The risk for consumers should preferably be estimated using a quantitative risk assessment approach, including dose response modelling, taking into account those factors that may vary considerably between countries (see also FAO/WHO, 2014 and Franssen et al., 2017).

When verification of controlled management is demonstrated by surveillance, absence of infection at a specific design prevalence (e.g., 1/1,000,000) may be used as an input into a Quantitative Risk Assessment Model to estimate the upper limit for the incidence of trichinellosis in consumers.

### 5.2 Implementing surveillance using slaughter samples

To apply a surveillance program to a population of pigs (e.g., pigs raised under conditions of controlled management), the population must be defined and all individuals in the population must be identifiable. The basic epidemiological unit is the pork production site or the compartment.

When an epidemiological unit of negligible risk pigs has been established, the number of pigs requiring testing must be determined based on the target design prevalence required to achieve the acceptable level of public health risk as described above. This number to be tested can be derived from various tables (e.g., Cannon and Roe, 1982).

When testing, it is important to perform sampling that is adequately representative of the pigs in the herd or compartment. Sampling design should follow recommendations promulgated by the World Organization for Animal Health (2018c), which include structured population-based surveys, such as systematic sampling at slaughter and random surveys.

### 5.3 Appropriate use of tests

Testing should be performed using suitably standardized and validated digestion or serological methods that are fit for purpose. There are various sources of information on appropriate methods and how these methods should be used in a quality assurance system. These references include:

• World Organization for Animal Health (2018) Manual of Diagnostic Tests and Vaccines for Terrestrial Animals.
• European Community (2015) Regulation (EC) No 2015/1375
• International Commission on Trichinellosis recommendations for quality assurance (QA) in digestion testing for Trichinella (on the ICT website (http://www.trichinellosis.org/) and in this Special Issue (Gajadhar et al., 2019).
• International Commission on Trichinellosis recommendations for serology on the ICT website (http://www.trichinellosis.org/) and in this Special Issue (Bruschi et al., 2019).
• ISO Standard 18743 (2015) Microbiology of the food chain – detection of *Trichinella* larvae in meat by artificial digestion method.

When using a test to determine prevalence, detailed knowledge of the performance characteristics of the test must be incorporated into the surveillance program. The performance of the test should be validated in the laboratory performing the test and an appropriate quality assurance system that includes proficiency testing should be in place.

## 6 Pigs from production sites not practicing controlled management

Where controlled management of domestic pigs is not implemented and routinely verified, individual carcass testing should be performed as described in the *ICT Quality Assurance in Digestion Testing Programs for* Trichinella (http://www.trichinellosis.org/Guidelines.html), and in Gajadhar et al. (2019) or pork should be subjected to post-slaughter treatment as described in the *ICT Recommendations on Post-harvest Control of* Trichinella *in Food Animals* (http://www.trichinellosis.org/Guidelines.html) and in Noeckler et al. (2019).

## Conflict of interest

The authors have no 'conflicts of interest.
